# Endoscopic inside stent placement is suitable as a bridging treatment for preoperative biliary tract cancer

**DOI:** 10.1186/s12876-015-0233-2

**Published:** 2015-02-05

**Authors:** Noritoshi Kobayashi, Seitaro Watanabe, Kunihiro Hosono, Kensuke Kubota, Atsushi Nakajima, Takashi Kaneko, Kazuya Sugimori, Motohiko Tokuhisa, Ayumu Goto, Ryutaro Mori, Koichi Taniguchi, Ryusei Matsuyama, Itaru Endo, Shin Maeda, Yasushi Ichikawa

**Affiliations:** 1 Medical Oncology Division, Yokohama City University School of Medicine, 3-9, Fuku-ura, Kanazawa-ku, Yokohama, 236-0004 Japan; 2 Gastroenterology Division, Yokohama City University School of Medicine, 3-9, Fuku-ura, Kanazawa-ku, Yokohama, 236-0004 Japan; 3 Gastroenterological Center, Yokohama City University Medical Center, Yokohama, Japan; 4 Gastroenterological Surgery Division, Yokohama City University School of Medicine, Yokohama, Japan

## Abstract

**Background:**

Endoscopic biliary stenting (EBS) is one of the most important palliative treatments for biliary tract cancer. However, reflux cholangitis arising from bacterial adherence to the inner wall of the stent must be avoided. We evaluated the use of EBS above the sphincter of Oddi to determine whether reflux cholangitis could be prevented in preoperative cases.

**Methods:**

Fifty-seven patients with primary biliary tract cancer were retrospectively recruited for the evaluation of stent placement either above (n = 25; inside stent group) or across (n = 32; conventional stent group) the sphincter of Oddi. We compared the stent patency periods prior to the time of surgical resection.

**Results:**

The preoperative periods were 96.3 days in the conventional stent group and 96.8 days in the inside stent group (*P* = 0.979). Obstructive jaundice and/or acute cholangitis occurred in 7 patients (28.0%) in the inside stent group and in 15 patients (46.9%) in the conventional stent group during the preoperative period (*P* = 0.150). The average patency periods of the stents were 85.2 days (range, 13–387 days) for the inside stent group and 49.1 days (range, 9–136 days) for the conventional stent group (log-rank test: *P* = 0.009). The mean numbers of re-interventions because of stent occlusion were 0.32 for the inside stent group and 1.03 for the conventional stent group (*P* = 0.026). Post-endoscopic retrograde cholangiopancreatography complications occurred in 2 patients in the inside stent group and 4 patients in the conventional stent group (*P* = 0.516). Postoperative liver abscess occurred in 1 patient in the inside stent group and 5 patients in the conventional stent group (*P* = 0.968). Inside stent placement was the only significant preventative factor associated with stent obstruction based on univariate (hazard ratio [HR], 0.286; 95% confidence interval [CI], 0.114-0.719; *P* = 0.008) and multivariate (HR, 0.292; 95% CI, 0.114-0.750; *P* = 0.011) analyses.

**Conclusion:**

Temporary plastic stent placement above the sphincter of Oddi is a better bridging treatment than conventional stent placement in preoperative primary biliary tract cancer.

## Background

Endoscopic biliary stenting (EBS) is a well-established palliative treatment that can be used to relive symptoms and improve quality of life of patients with biliary tract cancer [1,2]. EBS is also one of the most useful drainage methods in cases of primary biliary tract cancer accompanied by obstructive jaundice and/or cholangitis during the preoperative period [3]. However, the long-term placement of plastic biliary stents can itself become a cause of obstructive jaundice and/or reflux cholangitis. In addition, preoperative biliary drainage may be associated with an increased incidence of postoperative morbidity and lengthened hospital stays [4]. On the other hand, liver resection in jaundiced patients is associated with high morbidity and mortality rates [5]. There is still controversy regarding whether preoperative biliary drainage is essential for jaundiced patients with biliary tract cancer. Many Japanese studies report that postoperative mortality rates following major liver resection performed for hilar bile duct cancer are low, a finding that leads many to believe that preoperative biliary drainage an essential element in preoperative treatment [6,7]. Safe surgical resections cannot be performed in patients with reflux cholangitis, which is a life-threatening complication. Thus, preventing reflux cholangitis by performing endoscopic treatment is a challenge for the preoperative management of patients with biliary tract cancer. Recently, some reports have stated that an inside stent is very useful for preventing reflux cholangitis following liver transplantation [8,9]. Amsterdam-type or Tannenbaum-type fluorinated ethylene propylene endoprostheses occupying the entire bile duct can be used to prevent the reflux of intestinal contents into the biliary tree. To allow easy removal of the stent from the bile duct, a nylon thread is tied to the stent via a distal hole. Inside stents are known to be beneficial in patients with benign bile duct strictures, but only a small amount of evidence is available for cases of strictures associated with malignancies. According to a previous report, no significant difference in overall stent performance was observed between inside stents and conventional stents [10]. However, that previous study evaluated long patency periods of plastic stent placement lasting until the patient’s death and also excluded cases of malignant hilar biliary strictures. Recently, the administration of systemic chemotherapy and/or radiotherapy to patients with biliary tract cancer has been found to prolong both progression-free survival and overall survival [11]. Chemotherapy and/or radiotherapy are essential in patients with initially unresectable biliary tract cancer. However biliary tract infection is the most severe adverse event experienced by patients undergoing systemic chemotherapy for biliary tract cancer. This risk must be prevented to allow the safe continuation of systemic chemotherapy and/or radiotherapy. Some previous reports have found the patency period for plastic stents to be only a few months. If an inside stent could be used to prolong the patency period, chemotherapy and/or radiotherapy could be safely performed prior to surgical resection. In the present report, we retrospectively compared the use of conventional plastic stents and inside stents in patients with primary biliary tract cancer.

## Methods

### Patients

Eighty-two patients with malignant biliary strictures underwent surgical resected at Yokohama City University between 2006 and 2011. All patients had undergone computed tomography examinations, and they had been diagnosed with primary biliary tract cancer by an expert radiologist. We excluded 25 patients who had undergone percutaneous transhepatic biliary drainage (PTBD) as the initial treatment, and PTBD was performed following endoscopic treatment in this study. Fifty-seven patients were retrospectively enrolled in the present study. After evaluating the state of the patients (based on the presence of obstructive jaundice and/or acute cholangitis), endoscopic retrograde cholangiopancreatography (ERCP) was performed. All patients underwent performed surgical resection, and they were pathologically diagnosed with primary biliary tract cancer. We excluded cases of pancreatic cancer, ampullary cancer, and intra hepatic cancer (ICC). Most cases of pancreatic and ampullary cancer, are not indications for inside stent placement (the distance from the stricture to the papilla is less than 2 cm in most cases), and ICC is a separate entity from bile duct cancer according to surgical and pathological diagnosis, particularly in Japanese guidelines. In addition, many ICCs are characterized by expansive growth in the liver parenchyma, and there have been few cases of surgical resection following preoperative biliary drainage in our institution. According to resectability, we added preoperative chemotherapy or chemo-radiotherapy for the treatment of the patients with bile duct cancer. Twenty-seven patients (47.4%) underwent preoperative chemotherapy or chemo-radiotherapy. Patients received gemicitabine plus S-1 (gemcitabine 1,000 mg/m^2^ on days 1 and 8 plus S-1 60, 80, or 100 mg/d according to body surface area on days 1 through 14 of a 21-day cycle). When administered as neoadjuvant treatment, patients normally undergo at least three courses of this combination therapy. We re-evaluated resectability after three courses of chemotherapy, and if adverse events and disease progression were not observed, down- stage chemotherapy was continued until patients had received 12 courses. This study was performed retrospectively and was approved by our institution’s ethics committee (Yokohama City University Hospital, Clinical Research Approval No.185). The study was conducted in accordance with the principles of the Declaration of Helsinki.

### Preoperative endoscopic treatment

**Figure 1:**
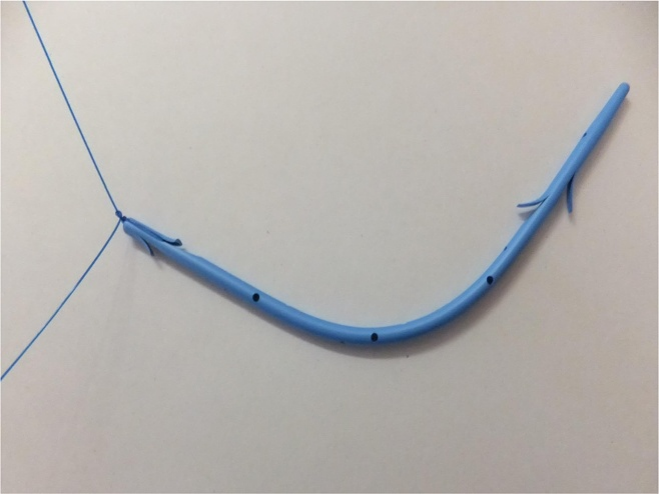
**Photograph of the Amsterdam type inside stent.** A 2–0 nylon thread is attached to the distal side hole to aid removal of the inside stent, when necessary.

**Figure 2:**
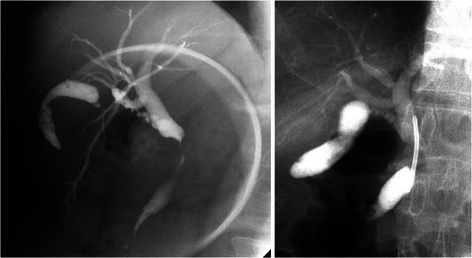
**Endoscopic cholangiography for a case of a Bismuth type I stricture.** An 8.5-Fr inside stent was placed above the sphincter of Oddi.

**Figure 3:**
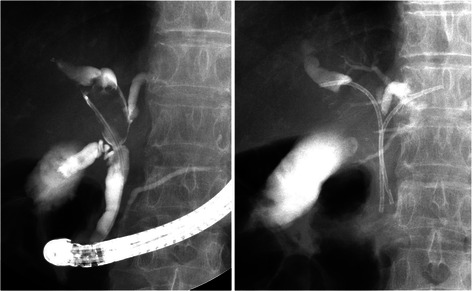
**Endoscopic cholangiography for a case of a Bismuth type IIIa stricture.** Two 7.0-Fr inside stents were placed above the sphincter of Oddi.

We evaluated the state of the patients and performed ERCP using a videoduodenoscope (JF260V or TJF260V; Olympus Medical Systems, Tokyo, Japan). The site and type of biliary strictures were confirmed by using cholangiography. The strictures were first negotiated with a 0.035- or 0.025-inch-diameter Jagwire (Microvasive Endoscopy; Boston Scientific Corp., Natick, MA, USA). If we had difficulties inserting the guide-wire through the stricture, we modified the placement technique by using a more hydrophilic guide-wire (Radifocus guide-wire; Terumo Co., Ltd., Tokyo, Japan). We inserted stents along the guide-wire through an endoscope. If the distance from the stricture to the sphincter of Oddi was at least 2 cm, we used a “conventional stent” (across the sphincter of Oddi) or an “inside stent” (above the sphincter of Oddi), and if the distance from the stricture to the sphincter of Oddi was less than 2 cm, we selected a conventional stent. The conventional stents used were 7-Fr Amsterdam-type polyethylene stents or 8.5-Fr or 10-Fr Tannenbaum-type Teflon stents (Cook Medical Inc., Bloomington, MN, USA), and the stent selection was at the discretion of each endoscopist. The inside stent were Amsterdam-type or Tannenbaum type fluorinated ethylene propylene endoprostheses (Cook Medical Inc.,) with a nylon thread tied to the stent via a distal hole (Figure 1) [9]. We also classified the biliary stricture site according to the Bismuth classification [12]. We used a single stent in the majority of cases, but 2 or 3 of the same type of stent (inside or conventional) were used in some cases with Bismuth type II or III strictures (Figures 2 and 3). An endoscopic nasobiliary drainage tube (ENBD tube) was used for the effective treatment of cholangitis and/or cytological or radiological analysis at the time of the initial endoscopic treatment in some cases. The stents were placed during the next session in these cases. An endoscopic sphinctectomy (EST) was additionally performed for the placement of 10-Fr stents. EST is a risk factor for reflux cholangitis, but we have experienced cases of severe acute pancreatitis following the placement of large caliber stents without EST. This procedure was not used for the placement of small caliber stents (7-Fr or 8.5-Fr). Following stent placement, many patients underwent preoperative chemotherapy and/or radiotherapy. If acute cholangitis and/or obstructive jaundice occurred during the preoperative period, emergency endoscopic treatment was immediately performed. We usually removed the stent and inserted an ENBD tube during emergency treatments. After a few days, a second stent of the same type was once again placed using the same technique as in the initial placement. If these endoscopic treatments were not successful, or jaundice/cholangitis did not improve even after endoscopic treatments, percutaneous transhepatic biliary drainage was performed as an additional treatment. We excluded these percutaneous treatment cases from our analysis, because this study evaluated the risk of cholangitis after preoperative endoscopic treatment. These treatments were repeated until the time of surgical resection.

### Evaluation

We evaluated the preoperative state of patients with or without cholangitis, and as well as the laboratory data at the time of the pre-endoscopic treatment. We defined the decompression period as the number of days from endoscopic treatment to attainment of a normal bilirubin level.

Stent occlusion was defined as recurrence of jaundice and/or evidence of dilated biliary systems detected by using some imaging modality. Proximal stent migration was defined as “located above the original stricture site” and distal migration was defined as “located below the original stricture site or no longer visible either endoscopically or radiographically”. We defined acute cholangitis as elevated levels of liver and/or biliary enzymes with the onset of fever (>38°C). We evaluated the number of endoscopic treatments until surgical resection. Post-ERCP complications were also evaluated according to Cotton’s criteria [13]. Additionally, we evaluated patients’ hospital stays after surgical resection and the occurrence of severe postoperative complications. In particular, we evaluated the presence of severe infectious complications (cholangitis that required the performance of percutaneous or endoscopic drainage, liver abscess that required the performance of percutaneous drainage or resulted in sepsis).

### Statistical analysis

The statistical analysis was performed using the Stat View program (SAS Institute, Cary, NC, USA). Categorical variables were compared using the *χ*^2^ test. Continuous variables were analyzed using the Student *t*-test. *P* values of less than 0.05 were considered statistically significant. The stent patency period was determined by the interval between stent insertion and occlusion or by the interval between stent insertion and surgical resection. Kaplan-Meier curves were constructed to describe the stent patency in each group. The stent patency periods were compared using a log-rank test. To analyze stent patency, patients without stent obstruction were censored at the time of analysis.

## Results

Fifty-seven patients were retrospectively enrolled in the current study. Twenty-five patients were pathologically diagnosed with Klatskin tumors, 30 with middle and lower bile duct cancers, and 2 with gallbladder cancers. All patients were followed-up until December 2012. The clinical features of the two groups were similar, as shown in Tables 1, 2 and 3. The laboratory data obtained before endoscopic treatment was not significantly different between the two groups (Table 4).

**Table 1:** **Clinicopathological characters of the patients with conventional stent**

Case	Disease	Bismuth	Preoperative treatment	Preoperative period (days)	Tumor size (mm)	Pathological grade	Stage JBC	Stage UICC
1	BD ca (Bim)	I		26	28	moderate	Stage IVa	Stage IIIa
2	BD ca (Bm)	I		58	12	well	Stage III	Stage IIIb
3	BD ca (Bim)	I		54	43	moderate	Stage III	Stage IIIb
4	BD ca (Bmi)	I		146	70	moderate	Stage III	Stage IIIb
5	BD ca (Bim)	I		49	60	well	Stage III	Stage IIIa
6	BD ca (Bi)	I		448	10	well	Stage III	Stage IIIb
7	BD ca (Bcls)	II	DSC + RT	105	25	well	Stage II	Stage II
8	BD ca (Bi)	I		40	17	well	Stage I	Stage I
9	BD ca (Bim)	I		40	22	moderate	Stage I	Stage I
10	BD ca (Bmi)	I		10	30	poor	Stage III	Stage I
11	BD ca (Bim)	I		77	46	well	Stage II	Stage I
12	BD ca (Bsm)	I		40	35	poor	Stage III	Stage IIIb
13	BD ca (Bim)	I		28	38	well	Stage III	Stage IIIa
14	BD ca (Bim)	I	RT	28	18	adenosquamous	Stage II	Stage II
15	BD ca (Bim)	II		90	30	moderate	Stage II	Stage IIIb
16	BD ca (Bmsc)	II	DSC	126	42	well	Stage II	Stage II
17	BD ca (Bcrls)	I	DSC + RT	217	55	moderate	Stage II	Stage II
18	BD ca (Bim)	I		43	90	poor	Stage III	Stage IIIb
19	BD ca (Bim)	I		45	28	well	Stage III	Stage IIIb
20	BD ca (Bmi)	I		37	55	well	Stage III	Stage IIIb
21	GB ca (C)	II	DSC	200	30	moderate	Stage III	Stage IIIa
22	BD ca (Bi)	I		22	28	moderate	Stage III	Stage IIIa
23	BD ca (BmC)	I		45	25	well	Stage II	Stage II
24	BD ca (Bmi)	I		20	68	well	Stage III	Stage IIIa
25	BD ca (Bs)	II	DSC	116	59	well	Stage IVb	Stage IIIa
26	BD ca (BmspC)	II	DSC	128	45	well	Stage I	Stage I
27	BD ca (BpcsmiJ)	IV	DSC	143	46	well	Stage III	Stage IIIb
28	BD ca (Brplcsm)	III	DSC	135	40	well	Stage IVa	Stage IVa
29	BD ca (Brcs)	III	DSC	105	30	well	Stage III	Stage IIIb
30	BD ca (Bcsrl)	III	DSC	114	50	moderate	Stage IVb	Stage IVa
31	BD ca (Bpcrls)	III	DSC	94	76	moderate	Stage IVa	Stage IVa
32	GB ca (GfbnC)	III	DSC	151	50	well	Stage III	Stage IIIa

*Abbreviations*: JSBS: Japanese Society of Biliary Surgery, UICC: Union for International Cancer Control, BD: bile duct, DSC: Down Stage Chemotherapy. RT: radiotherapy.

**Table 2:** **Clinicopathological characters of the patients with inside stent**

Case	Disease	Bismuth	Preoperative treatment	Preoperative period (days)	Tumor size (mm)	Pathological grade	Stage JBC	Stage UICC
1	BD ca (Bm)	I		27	36	moderate	Stage III	Stage IIIb
2	BD ca (Bm)	I		36	48	moderate	Stage III	Stage IIIb
3	BD ca (Bmi)	I		55	32	moderate	Stage III	Stage IIIb
4	BD ca (Bms)	I		50	35	poor	Stage III	Stage IIIb
5	BD ca (BmjiC)	I		69	32	moderate	Stage III	Stage IIIb
6	BD ca (Bsmlrc)	II		34	65	poor	Stage III	Stage IIIb
7	BD ca (Blcrsm)	II		44	24	poor	Stage III	Stage IIIb
8	BD Ca (Bsmc)	II	DSC	124	35	moderate	Stage III	Stage IIIb
9	BD ca (Bsmc)	II	DSC	114	35	adenosquamous	Stage I	Stage I
10	BD ca (Bim)	I	DSC	43	24	poor	Stage III	Stage IIIb
11	BD ca (Bmi)	I		120	62	moderate	Stage III	Stage IIIb
12	BD ca (Bmi)	I		30	25	moderate	Stage I	Stage I
13	BD ca (Bs)	II	DSC	141	25	moderate	Stage I	Stage I
14	BD ca (Bm)	I		14	14	well	Stage II	Stage II
15	BD ca (Bp)	IV	DSC	169	36	moderate	Stage III	Stage IIIb
16	BD ca (Bmsi)	II		50	39	moderate	Stage II	Stage II
17	BD ca (Bims)	II	DSC	140	90	well	Stage III	Stage IIIb
18	BD ca (Bsm)	III	DSC	117	40	well	Stage II	Stage II
19	BD ca (Bsclr)	III	DSC	116	55	moderate	Stage II	Stage II
20	GB ca (CGn)	I	DSC	142	67	moderate	Stage III	Stage IIIb
21	BD ca (Brcs)	III	DSC	29	30	well	Stage III	Stage IIIb
22	BD ca (Bs)	III	DSC	135	6	well	Stage I	Stage I
23	BD ca (Bsm)	II	DSC	387	23	well	Stage I	Stage I
24	BD ca (Bms)	I	DSC	107	25	moderate	Stage I	Stage I
25	BD ca (Brcsl)	II	DSC	110	45	poor	Stage IVa	Stage IVa

*Abbreviations*: JSBS: Japanese Society of Biliary Surgery, UICC: Union for International Cancer Control, BD: bile duct, DSC: Down Stage Chemotherapy. RT: radiotherapy.

**Table 3:** **Clinicopathological features of both groups**

	Age median (range)	Sex (M/F)	Stage (JSBS) (I/II/III/ IVa/IVb)	Stage (UICC) (I/II/IIIa/IIIb/IVa/IVb)	Bismuth classification (I/II/III/IV)	Tumor size median (cm) (range)	Pathological diagnosis (well/moderate/poor/other)	Preoperative chemotherapy (yes/no)
Conventional stent group	70.5 (56–86)	26/6	3/6/18/3/2	4/5/7/13/3/0	20/6/5/1	39.0 (10–90)	17/11/3/1	13/19
Inside stent group	71.0 (57–84)	19/6	6/4/14/1/0	6/4/2/12/1/0	11/9/4/1	35.0 (6–90)	6/13/5/1	14/11
P value	0.378	0.732	0.817	0.453	0.510	0.612	0.320	0.155

*Abbreviations*: JSBS: Japanese Society of Biliary Surgery, UICC: Union for International Cancer Control.

**Table 4:** **Laboratory data before the endoscopic treatment of both groups**

	T-Bil (mg/dl) average (range)	AST (IU/ml) average (range)	ALT (IU/ml) average (range)	ALP (IU/ml)	WBC (/μl)	CRP (mg/dl)	CA19-9 (U/ml)	CEA (ng/ml)	Initial endoscopic treatment (infection: yes/no)
Conventional stent group	6.40	148.5	216.5	1448.8	7233.7	3.54	326.7	3.29	15/17
Inside stent group	5.60	123.0	139.1	1279.1	8126.7	2.04	335.7	3.01	7/18
P value	0.631	0.534	0.201	0.621	0.520	0.356	0.965	0.621	0.706

*Abbreviations*: CA19-9: Carbohydrate antigen 19–9, CEA: Carcinoembryonic antigen.

The clinical features at the time of the preoperative endoscopic treatment are shown in Table 5. Eleven patients received ENBD tube placement at the time of initial drainage; 7 of these had acute cholangitis. In these cases, an inside stent or a conventional stent was put in position during the next endoscopic treatment. At the time of the initial treatment, post-ERCP complications occurred in 6 patients (4 cases of mild pancreatitis in the conventional stent group and 1 case of mild pancreatitis and 1 case of moderate bleeding in the inside stent group; *P* = 0.516). Stent migration occurred in 2 patients in the inside stent group (1 case on the proximal side and 1 case in the distal side; *P* = 0.979); stent migration did not occur in any patients in the conventional stent group. All the patients’ symptoms were alleviated following stent placement, and the laboratory data normalized soon thereafter. The total bilirubin level normalized at 18.0 days in the conventional stent group and at 12.9 days in the inside stent group (*P* = 0.438). Stent diameters of 7, 8.5, and 10 Fr were used in 26, 3, and 3 patients in the conventional stent group and in 9, 13, and 3 patients in the inside stent group, respectively (*P* = 0.820). One, two, and three stents were used in 24, 6, and 2 patients in the conventional stent group and in 23, 2, and 0 patients in the inside stent group, respectively (*P* = 0.283).

**Table 5:** **Preoperative endoscopic treatment of both groups**

	Preoperative periods (days) median (range)	Decompressi on periods (days) average (range)	Stent patency Periods (days) average (range)	Stent obstruction case (percentage)	Re-intervention by obstruction times (average)	Stent diameter (7Fr/8.5 F r/10Fr)	Stent number (1/2/3)
Conventional stent group	96.3 (10–448)	18.0 (1–81)	49.1 (9–136)	15 (46.9%)	1.03	26/3/3	24/6/2
Inside stent group	96.8 (14–387)	12.9 (1–36)	85.2 (13–387)	7 (28%)	0.32	9/13/3	23/2/0
P value	0.979	0.438	0.009	0.150	0.026	0.820	0.283

**Figure 4:**
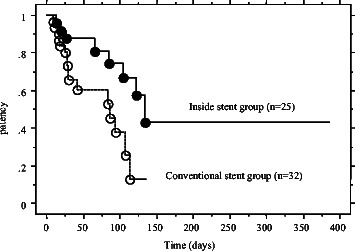
**Kaplan-Meier curve of patency of the stent for the two groups.** There was a statistically significant difference in the stent patency period between the groups (*P* = 0.009).

The preoperative periods were nearly the same in the two groups (96.3 days vs. 96.8 days). The 50% patency periods for the stents were 49.1 days in the conventional stent group and 85.2 days in the inside stent group (*P* = 0.009) (Figure 4). Obstructive jaundice and/or acute cholangitis occurred during the preoperative period in 15 patients (46.9%) in the conventional stent group and in 7 patients (28%) in the inside stent group (*P* = 0.150). The average number of re-interventions was 1.9 in the conventional stent group and 0.32 in the inside stent group (*P* = 0.026).

All patients underwent surgical resection (pancreaticoduodenectomy, n = 33; extrahepatic bile duct resection, n = 1; pancreaticoduodenectomy with hepatectomy, n = 1; hepatectomy, n = 22; Table 6). The length of hospital stay was 37.5 days in the conventional stent group and 41.7 days in the inside stent group (*P* = 0.560). Postoperative complications occurred in 17 patients. The morbidity rates were 34.3% (11/32) in the conventional stent group and 24.0% (6/25) in the inside stent group. A pancreatic fistula occurred in 4 patients (2 cases in the conventional stent group and 2 cases in the inside stent group), and a biliary fistula occurred in 3 patients (1 case in the conventional stent group and 2 cases in the inside stent group). Liver abscess that might be treated with percutaneous drainage occurred in 5 patients in the conventional stent group and in 1 patient in the inside stent group (*P* = 0.968). The mortality rates were 0% (0/32) in the conventional stent group and 4% (1/25) in the inside stent group. The single hospital death occurred because of active bleeding from a hepatic artery aneurysm in the inside stent group.

**Table 6:** **Post-ERCP and Post-operative complications and operative procedure of both groups**

	Post ERCP complication (Pancreatitis/Cholangitis/Bleeding)	Stent migration	Operation (PD/EHBD/HR+ EHBD/HPD)	Hospital stay (day)	Postoperative complication (total)	Abscess
**Conventional stent group**	**(4/0/0)**	**0 (0%)**	**(20/1/11/0)**	**37.5 (13–114)**	**11 (34.3%)**	**5 (15.6%)**
Inside stent Group	(1/0/1)	2 (8%)	(13/0/11/1)	41.7 (17–131)	6 (24.0%)	1 (4%)
P value	0.516	0.979	0.0783	0.560	0.3101	0.968

PD: pancreaticoduodenectomy, EHBD: extrahepatic bile duct resection, HR: hepatectomy HPD: pancreaticoduodenectomy with hepatectomy.

We analyzed some of the factors associated with stent obstruction. We describe only a few important factors related to stent obstruction (*P* < 0.2) in Table 7. Inside stent was the only significant preventative factor associated with stent obstruction based on univariate (hazard ratio [HR], 0.286; 95% confidence interval [CI], 0.114 -0.719; *P* = 0.008) and multivariate (HR, 0.292; 95% CI, 0.114 -0.750; P = 0.011) analyses.

**Table 7:** **Univariate and multivariate analysis of stent obstruction**

	Univariate analysis			Multivariate analysis		
	Hazard ratio	95% confidence interval	P value	Hazard ratio	95% confidence interval	P value
Inside stent	0.286	0.114-0.719	0.008	0.292	0.114-0.750	0.011
Preoperative chemotherapy (Yes)	2.254	0.813-6.249	0.118	1.614	0.579-4.499	0.360
Initial endoscopic treatment with cholangitis	2.267	0.741-6.934	0.151	2.530	0.807-7.934	0.111
Stent diameter 7Fr.	1.106	0.372-3.289	0.8558			

## Discussion

The use of inside stents in biliary stricture cases was first reported in 1991 [10]. For benign stricture cases, in particular, excellent outcomes were reported [8,9]. Hisatsune reported that EBS appeared to be efficacious for the treatment of multibranched post-transplantation biliary strictures [9]. As many as three inside stents can be placed without requiring an endoscopic sphincterotomy because the distal ends of these stents do not compress the orifice of the pancreatic duct. The endoscopic placement of multiple biliary stents usually requires a sphincterotomy because the duodenal ends of the stents compress the orifice of the pancreatic duct, which sometimes leads to acute pancreatitis. In 2012, Moon reported on the use of a modified fully covered self-expandable metal stent [14]. The metallic stents were put in place for the temporary treatment of benign biliary strictures above the ampulla of Vater and removed after 3 to 5 months. Technical success and improvement in ductal strictures were achieved for almost all the cases, and stent-induced bile duct injuries were not observed.

On the other hand, evidence that an inside stent is more useful than a conventional stent has remained inadequate for malignant biliary strictures. To our knowledge, only one prospective, randomized clinical study comparing the utility of stents placed above and across the intact sphincter of Oddi has been performed, by Pedersen et al. [10]. In this previous study, the median stent function time did not differ between the two groups. However, significantly more patients in the inside stent group experienced stent migration. After the publication of Pedersen’s findings, some retrospective studies were also reported. Uchida assessed 16 patients with malignant biliary obstructions in whom a stent was placed above an intact sphincter of Oddi [15]. The median patency period was significantly longer than that for conventional stents. In their study, most of the patients had pancreatic cancer. However, in many patients with inoperative malignant biliary strictures, a self-expanding metallic stent was selected to enable a longer patency period than that for a plastic stent [16]. Unfortunately, preoperative metallic stent placement creates an obstacle for subsequent surgical resection. Particularly in cases of hilar bile duct cancer, the use of a metallic stent makes subsequent resection very difficult and risky [17]. Thus, an inside stent seems to be a very suitable choice for preoperative stent placement.

In this study, we selected only patients with primary biliary tract cancer for the following reasons. First, many patients with distal biliary strictures have pancreatic or ampullary cancer, and the placement of an inside stent in the biliary stricture near the distal end can be difficult in these cases. According to past reports, the length between the lower end of the stricture and the sphincter was more than 2 cm in all cases of hilar biliary cancer, as well as in two-thirds of non-hilar biliary cancer cases, while it was less than 1 cm for most pancreatic cancers [18]. In our study, we selected only patients in whom the distance to the lower end of the stricture was more than 2 cm from the sphincter. Second, pancreatic cancer in the head of the pancreas is associated with a different type of damage to the bile epithelial cells, compared with primary biliary tract cancer. Bile duct epithelial dysplasia and fibrosis are secondary occurrences in patients with pancreatic cancer. Third, cancer of the head of the pancreas frequently involves duodenal and perineural invasion. Duodenal movements and/or duodenal luminal strictures are very important factors in reflux cholangitis.

In our study, the use of an inside stent prolonged the patency period by approximately 3 months. Recently, downstage chemotherapy has been adopted by some institutions [19], and the safe performance of preoperative chemotherapy or radiotherapy without cholangitis is very important for these treatments. An inside stent was more useful and suitable than a conventional stent for the completion of these preoperative treatments. In some previous reports, an ENBD tube was reported to be a suitable form of drainage for hilar bile duct cancer [20]. However, nasobiliary tube placement cannot be endured for 2 or 3 months because of the development of nasal ulcers, and some cases were located at the esophagogastric junction. The long-term placement of an ENBD tube is also not suitable for patients undergoing preoperative chemotherapy or radiotherapy.

Many patients in the inside stent group did not require re-intervention before surgical treatment. Stent migration occurred in only 2 patients in the inside stent group. According to previous reports, stent migration occurred significantly more frequently with an inside stent than with a conventional stent [10]. In the present study, we did not cut a flap at the proximal end for the inside stent, and such a flap might have anchored the stent to the biliary epithelium. There were some cases of obstructive jaundice with acute cholangitis (n = 5) and without acute cholangitis (n = 2) in the inside stent group. On the other hand, all the patients experienced acute cholangitis and required emergency endoscopic treatment in the conventional stent group. Even if a stent obstruction occurred with an inside stent, reflux cholangitis might occur smoothly compared with a conventional stent. Of course, if obstructive jaundice and/or acute cholangitis did happen, we might remove an inside stent more carefully than a conventional stent because of the risk that the distal end of the stent could cause injury to the biliary epithelial walls.

Post-ERCP complications did not differ significantly between the two groups. In most cases, we did not perform a sphinctectomy, and the inside stent was easily positioned in many of the cases. The papilla of Vater was not subjected to severe damage from the use of an inside stent, compared with the use of a conventional stent. The conventional stent was associated with a higher tendency of pancreatic duct obstruction, compared with the inside stent. In our study, bleeding occurred in 1 patient in the inside stent group. This patient experienced an injury at the esophagogastric junction at the time of the removal of the inside stent. This complication did not seem to be a specific accident arising from the use of an inside stent.

Preoperative cholangitis is reportedly a risk factor for hepatic insufficiency following major hepatic resection [21]. Sakata et al. evaluated the effects of preoperative cholangitis on in-hospital mortality among patients with hilar cholangiocarcinoma. They found that the mortality rate was higher among patients with cholangitis than among those without this complication, and cholangitis was the only independent variable related to postoperative in-hospital mortality. In the current study, only 1 case of severe infection occurred after surgical resection in the inside stent group. With regard to this point, the use of an inside stent might be suitable for the prevention of postoperative complications.

We also analyzed some of the important factors related to stent obstruction. EST, Bismuth classification, and stent diameter were not significant preventative factors associated with stent obstruction in this study (data not shown). We should re-analyze these factors in a large number of patients, but it is very important that according to our findings, inside stent was the only significant preventative factor.

This study was not a randomized study, and the skill of the individual endoscopist might have differed slightly between the two groups. However, all the treatments were performed by the same three expert endoscopists (with 20, 16, and 15 years of experience, respectively). Thus, any technical bias is likely to be very small in this study. However, there is some selection bias in this study. Patient selection bias (obstructive jaundice with cholangitis in initial treatment, chemotherapy before surgical resection, stent diameter, stent length, among others) could not rule out in this retrospective study.

## Conclusion

In summary, the endoscopic treatment of biliary strictures using an inside stent is very useful and safe. Furthermore, the stent patency of inside stent was significantly longer than that of conventional stents. In addition, the incidence of acute cholangitis and the need for reintervention or emergency intervention were notably lower in the inside stent group. Post-ERCP and postoperative complications did not differ significantly between the two groups. We propose that the use of an inside stent as a temporary preoperative stent might be useful in patients with primary biliary tract cancer.
